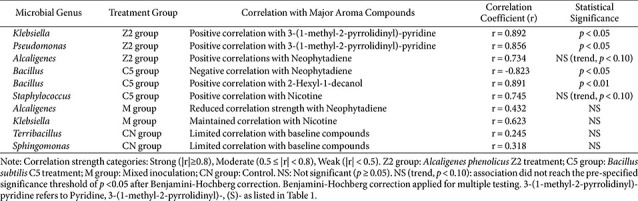# Differential Enhancement of Cigar Tobacco Leaf Aroma by Single-Strain Inoculation with *Alcaligenes phenolicus* and *Bacillus subtilis*

**DOI:** 10.4014/jmb.2601.01012

**Published:** 2026-05-12

**Authors:** Fang Liu, Linan Xu, Miao Lai, Zhishun Chai, Mingqin Zhao

**Affiliations:** 1 Flavors and Fragrance Engineering & Technology Research Center of Henan Province, College of Tobacco Science, Henan Agricultural University, Zhengzhou 450046, P. R. China; 2 Cigar Fermentation Technology Key Laboratory of China Tobacco, China Tobacco Sichuan Industrial Co., Ltd., China Tobacco Technology Innovation Center for Cigar, Chengdu 610101, P. R. China

**Keywords:** Cigar tobacco leaves, Aroma-producing strain, Microbial fermentation, Aroma compounds, Microbial interaction

## Abstract

Flavor deficiency in cigar tobacco leaves (CTLs) limits cigar industry development. This study isolated two aroma-producing bacteria (*Alcaligenes phenolicus* Z2 and *Bacillus subtilis* C5) from CTLs during air-curing. Single strain inoculation resulted in treatment-specific aroma compound changes: Z2 treatment was associated with enhanced diterpene compounds (notably neophytadiene and 1-heptacosanol), while C5 markedly increased fatty alcohol concentrations (2-hexyl-1-decanol, 9.67-fold) and nicotine levels. Mixed inoculation generally showed intermediate rather than synergistic effects. Variable importance in projection (VIP) analysis identified nicotine, neophytadiene, and nonadecatrienediol as the most discriminatory compounds among treatments. Microbial community analysis revealed that exogenous strain inoculation significantly altered indigenous microbiota composition and interaction patterns, with Z2 treatment associated with positive microbial co-occurrence patterns, while mixed inoculation displayed network characteristics suggestive of increased competitive interactions. Correlation analysis revealed strain-specific microbial-metabolite associations, though causal relationships require further validation. These findings suggest that single strain inoculation produces distinct metabolic profiles with potential for strain-specific tobacco aroma modulation.

## Introduction

As a high-end tobacco product, cigars have experienced continuous growth in global markets, with the international cigar and cigarillos market projected to reach USD 120.71 billion by 2034 [1-2]. The growing global demand reflects increasing consumer interest in premium tobacco products, though this expansion brings challenges regarding product quality and safety considerations. Recent comprehensive meta-analyses involving approximately 24,000 adults have highlighted the importance of understanding tobacco product composition and its health implications, emphasizing the need for improved manufacturing processes [3]. Since 2015, China's cigar industry has expanded rapidly at an annual rate exceeding 40%, representing a significant growth sector in the tobacco industry [4].

Ecological conditions and production techniques often result in cigar tobacco leaves with indistinct style characteristics and suboptimal aroma quality across different geographic regions, creating universal needs for flavor profile improvements. Recent studies on tobacco flavor enhancement have focused on cooling flavors and flavor compounds across various tobacco products, highlighting the universal importance of aroma quality in premium tobacco markets [5]. Consumer preference studies have demonstrated that flavor characteristics significantly influence product selection and usage patterns, with longitudinal research showing associations between flavored tobacco products and consumer behavior across different demographics [6]. During cigar production, fermentation constitutes a critical process influencing characteristic flavor development and final product quality worldwide [7-8]. Traditional approaches optimizing fermentation through temperature, humidity, and pH modifications have shown limited success globally [9-10], highlighting the need for alternative strategies to enhance cigar tobacco leaf aroma quality regardless of production origin.

Recently, utilizing aroma-producing microorganisms for tobacco leaf fermentation has attracted considerable international research interest. The complex chemical composition of tobacco leaves and nicotine presence create universal challenges for non-tobacco microorganisms to successfully colonize cigar tobacco leaves, regardless of variety or geographic origin. Global cigar tobacco processing operations during curing harbor diverse microbial communities, potentially providing suitable sources for screening adapted aroma-producing microorganisms with applications across different tobacco cultivars and production systems [11].

Effective tobacco fermentation likely depends on both the characteristics of added microorganisms and their interactions with indigenous microbial communities—a principle that applies universally across cigar tobacco production systems worldwide [12]. Current international research emphasizes microbial effects on tobacco leaf chemical composition, with limited investigation of impacts on existing microbial communities and interaction mechanisms across different tobacco varieties and processing conditions [13]. Understanding how introduced exogenous microorganisms influence cigar tobacco leaf microbial community structure and their potential interactions with existing populations represents a crucial research need for applying biofermentation technology in consistent high-aroma cigar tobacco leaf production globally, with implications for premium tobacco markets worldwide.

## Materials and Methods

### Experimental Materials and Culture Media Preparation

CX-4 cigar tobacco leaves (CTLs) from Dazhou, Sichuan Province, China served as research subjects. All chemical reagents were analytical grade from China National Pharmaceutical Group. Luria-Bertani medium contained tryptone (10 g/L), yeast extract (5 g/L), sodium chloride (10 g/L), pH 7.0-7.4, sterilized at 121°C for 20 min. Fermentation medium (FM) utilized a modified tobacco water extract method: 15 g tobacco samples were boiled in 350 mL distilled water for 30 minutes, filtered through four layers of gauze; the final medium combined 20 mL tobacco extract with 2 g glucose and 80 mL distilled water, sterilized at 115°C for 30 min. Total soluble sugar and nitrogen content were measured spectrophotometrically using standard protocols to ensure batch-to-batch consistency (coefficient of variation <5%).

### Screening and Isolation Techniques for Aroma-Producing Strains

Air-cured CTLs were used for isolation of aroma-producing strains following established microbiological protocols. Ten grams of aseptically processed tobacco leaves were shaken at 200 rpm in 90 mL sterile physiological saline for 60 minutes at 30°C. Serial dilutions (10^-1^–10^-5^) were prepared, with 100 μL spread on FM solid medium and incubated at 37°C for 24 h. Although initial screening was conducted at 37°C to maximize colony recovery, subsequent fermentation experiments were performed at 30°C to reflect practical cigar tobacco fermentation conditions. Single colonies were purified by streak plate method and evaluated using a standardized olfactory assessment by 10 professionally trained evaluators (5 males, 5 females, aged 22–40) following sensory evaluation protocols. Evaluators underwent standardized training using reference aroma standards prior to assessment. Evaluation criteria included aroma intensity, type, persistence, and overall acceptability scored on a 5-point scale. Strains scoring ≥3.5 with distinctive characteristics were selected for further analysis, with negative controls and blind evaluation procedures ensuring objective assessment.

The establishment of inoculated strains during fermentation was assessed through relative abundance changes of Alcaligenes and Bacillus genera in 16S rRNA amplicon sequencing data, serving as indirect indicators of inoculated strain persistence. Direct quantitative tracking via time-series sampling was not performed, which represents a limitation acknowledged in this study.

### Molecular Identification Methods of Strains

Selected aroma-producing strains underwent molecular identification using 16S rRNA gene sequence analysis according to standard taxonomic protocols. Genomic DNA was extracted using E.Z.N.A.^®^ Soil DNA Kit (Omega Bio-tek, USA) following manufacturer's protocols. PCR amplification employed universal bacterial primers 27F (5'-AGTTTGATCMTGGCTCAG-3') and 1492R (5'-GCTTACCTTGTTA CGACTT-3') in a 50 μL reaction system. PCR conditions included initial denaturation (95°C, 5 min); 35 cycles of denaturation (95°C, 30 s), annealing (55°C, 30 s), extension (72°C, 90 s); and final extension (72°C, 10 min). Products were verified by 1.5% agarose gel electrophoresis before sequencing (Tsingke Biotechnology, China). Sequences were analyzed using NCBI BLAST database comparison, and phylogenetic trees constructed using MEGA X software based on the neighbor-joining method with 1000 bootstrap repetitions for statistical support assessment.

### Biological Fermentation Process Design for Cigar Tobacco Leaves

The experimental design comprised four treatment groups using a completely randomized design: Z2 single strain inoculation, C5 single strain inoculation, Z2 + C5 mixed inoculation (M group), and uninoculated control (CN). Each treatment included three biological replicates to enable statistical analysis. Although three biological replicates per treatment represent a borderline sample size for multivariate analyses, this design is consistent with published tobacco fermentation studies. Where technical replicates were performed for GC-MS analysis, values were averaged prior to statistical testing. Statistical robustness was supported through stringent analytical thresholds: PERMANOVA with 999 permutations, Benjamini-Hochberg FDR correction for multiple comparisons, and conservative correlation thresholds (|ρ| ≥ 0.6) for network construction.

Strains cultured on LB agar were transferred to solid FM medium and incubated at 30°C for 24 h to ensure active growth phase. Bacterial suspensions were prepared at standardized 1 × 10^7^ CFU/mL concentration verified by plate counting method. For single-strain treatments, tobacco leaves were mixed with inoculant at 15% (v/w); for mixed inoculation, equal volumes of Z2 and C5 suspensions were combined maintaining total inoculum volume. All treatments underwent controlled fermentation at 30 ± 1°C with 80 ± 5% relative humidity for 14 days. The 14-day fermentation period was selected based on previously reported cigar tobacco fermentation protocols [14], which identified this duration as representative of active fermentation stages. The single time-point sampling design represents a limitation of this study, as temporal dynamics of strain activity and metabolite accumulation could not be fully captured. Standardized pile turning was performed every 6 hours to ensure uniform conditions. Post-fermentation samples were immediately flash-frozen in liquid nitrogen, ground to fine powder using sterile procedures, and stored at -80°C until analysis. Control groups received equivalent volumes of sterile distilled water. Throughout fermentation, environmental parameters (temperature, moisture content) were monitored at 24-h intervals to verify consistent conditions across all treatment replicates.

The persistence of inoculated strains during fermentation was indirectly assessed through relative abundance changes of Alcaligenes and Bacillus genera in 16S rRNA amplicon sequencing data obtained from post-fermentation samples. Direct quantitative tracking via time-series sampling was not performed, which represents a limitation acknowledged in this study.

### Analysis of Aroma Components by Gas Chromatography-Mass Spectrometry

Volatile aroma compounds in cigar tobacco leaves were analyzed using validated automated headspace solid-phase microextraction comprehensive two-dimensional gas chromatography-mass spectrometry (HS-SPME-GC×GC-MS) methodology. Dried (40°C, 24 h) and ground tobacco samples (1.000±0.001 g) were precisely weighed into 20 mL amber extraction bottles and hermetically sealed. The Smart SPME fiber (DVB/C-WR/PDMS 80/10 μm, Supelco) was conditioned at 250°C for 30 min in the GC-MS injector prior to each analysis to ensure reproducible performance.

Sample preparation followed standardized protocols: preheating at 85°C for 15 min to establish equilibrium, followed by headspace extraction for 50 min under agitation (500 rpm), and thermal desorption at 250°C for 5 min. Quality control blanks and standard reference materials were included in each analytical batch to monitor system performance and potential contamination.

Chromatographic separation utilized a validated two-column system: first-dimension DB-Wax column (30 m × 0.25 mm × 0.25 μm, Agilent Technologies) for polar compound separation and second-dimension DB-17ms column (1.2 m × 0.18 mm × 0.18 μm, Agilent Technologies) for enhanced resolution. The optimized temperature program consisted of: initial temperature 40°C (hold 5 min), ramped at 5°C/min to 240°C (hold 10 min), total run time 55 minutes. Ultra-high purity helium carrier gas (99.9999%) flowed at 1.0 mL/min in splitless mode with injector temperature maintained at 250 ± 1°C.

Comprehensive two-dimensional separation employed a solid-state thermal modulator (Zoex Corporation) with nitrogen as cryogenic gas, utilizing HV series modulation column C720 with 6-second modulation period optimized for volatile compound analysis. Modulator inlet and outlet temperatures tracked the primary GC oven program with ±2°C precision, while the cold trap was maintained at -50 ± 2°C throughout analysis.

Mass spectrometry detection parameters were optimized for volatile organic compound analysis: electron impact ionization (70 eV), ion source temperature 280°C, transfer line temperature 250°C, and mass range 41-500 m/z acquired in full scan mode with 0.05-second scan interval and 20,000 amu/sec scan rate. Solvent delay was set at 5.0 min to protect the detector filament.

### Data Processing and Compound Identification

Compound identification employed rigorous criteria: retention time and mass spectral matching against NIST20 database with minimum match quality >800 (80% confidence). Peak integration quality control excluded peaks with signal-to-noise ratio <3, relative standard deviation >30.0% across technical replicates, or area percentage <0.01% of total chromatogram. Relative quantification utilized peak area normalization with internal standard correction when available.

All samples underwent triplicate analysis with technical replicates averaged prior to statistical testing to calculate final values. Method blanks, procedural blanks, and quality control standards were analyzed with each sample batch to ensure data integrity. Complete data for all 63 detected volatile compounds, including retention times, mass spectral match scores, and relative abundances across all treatment groups, are provided in Supplementary Table S1.

The 20 major aroma compounds presented in Table 1 were selected using the following pre-specified multivariate statistical criteria: (1) statistical significance (*p* < 0.05) determined by one-way ANOVA with Tukey's HSD post-hoc tests, (2) variable importance in projection scores from PLS-DA analysis, prioritizing VIP ≥ 1.0 compounds, and (3) chemical class representation to ensure comprehensive coverage of tobacco aroma-relevant compound categories, including alkaloids, diterpenes, fatty alcohols, terpenoids, fatty acid esters, alkenes, ketones, furanones, and other organic compounds previously reported in tobacco flavor literature. These criteria were defined prior to data analysis to minimize selection bias. Multiple comparison corrections using the Benjamini-Hochberg false discovery rate procedure (α = 0.05) were applied to control Type I error inflation, with adjusted p-values reported for statistical robustness.

### Microbiome Diversity Sequencing Analysis

Microbial genomic DNA was extracted from 15 g tobacco leaf samples homogenized in liquid nitrogen using the E.Z.N.A. Soil DNA Kit (Omega Bio-tek, Norcross, USA) following manufacturer's protocols with minor modifications for plant tissue. DNA integrity was assessed by 1% agarose gel electrophoresis and purity evaluated using A260/A280 and A260/A230 ratios. DNA concentration was quantified using a NanoDrop 2000 spectrophotometer (Thermo Fisher Scientific, USA) with acceptable concentrations >20 ng/μL and purity ratios between 1.8-2.0.

For bacterial community profiling, the hypervariable V5–V7 region of the 16S rRNA gene was amplified using primers 799F (5'-AACMGGATTAGATACCCKG-3') and 1193R (5'-ACGTCATCCCCACCTTCC-3') in triplicate PCR reactions to minimize amplification bias. The V5–V7 region was selected in conjunction with the 799F primer, which was specifically designed to minimize co-amplification of plant plastid (chloroplast and mitochondrial) 16S rRNA sequences — a critical consideration for plant-derived samples such as tobacco leaves, where chloroplast sequences can dominate amplicon libraries when conventional V3–V4 primers are employed. This primer combination provides reliable taxonomic resolution while effectively reducing plant-derived sequence interference in tobacco leaf microbiome profiling.Fungal community analysis targeted the internal transcribed spacer (ITS) region using primers ITS1F (5'-CTTGGTCATTTAGAGGAAGTAA-3') and ITS2R (5'-GCTGCGTTCTTCATCGATGC-3'). PCR conditions included initial denaturation (95°C, 3 min), 35 cycles of denaturation (95°C, 30 s), annealing (55°C, 30 s), extension (72°C, 45 s), and final extension (72°C, 10 min). Amplicon quality was verified by 2% agarose gel electrophoresis, and products were purified using AxyPrep DNA Gel Extraction Kit (Axygen Biosciences, USA). Equal amounts of purified amplicons were pooled and sequenced using the Illumina MiSeq platform (2 × 300 bp paired-end) at Major Bio-Pharm Technology Co., Ltd., (China) following standard protocols.

### Bioinformatics and Statistical Analysis Methods

Raw sequencing reads underwent quality control using FASTP software with adapter trimming and quality filtering (Q20 threshold). Paired-end reads were merged using FLASH software, and chimeric sequences removed using UCHIME algorithm. Operational taxonomic units (OTUs) were clustered at 97% similarity using UPARSE pipeline, with taxonomic assignment performed against SILVA database (release 138) for bacteria and UNITE database (version 8.2) for fungi.

Aroma compound data visualization employed hierarchical clustering heat maps generated using TBtools software (version 1.1043) based on Euclidean distance matrices and Ward's clustering algorithm. Color intensity in the heat map represents log-transformed and z-score normalized relative peak areas to enable visual comparison across compounds with different abundance ranges. The analytical framework incorporated comprehensive GC-MS data from all 63 identified compounds across treatment groups to ensure complete representation of chemical diversity patterns.

Microbial community structure analysis utilized several complementary approaches: (1) alpha diversity metrics including Shannon, Simpson, and Chao1 indices calculated using QIIME2 platform, (2) beta diversity assessment through principal coordinate analysis (PCoA) using Bray-Curtis dissimilarity matrices, and (3) statistical significance testing via permutational multivariate analysis of variance (PERMANOVA) with 999 permutations to evaluate treatment effects on community composition. Given the limited sample size of three biological replicates per treatment, conservative analytical thresholds were applied throughout: Benjamini-Hochberg FDR correction for all multiple comparisons, and stringent correlation thresholds (|ρ| ≥ 0.6) for network construction to minimize spurious associations.

Microbial co-occurrence networks were constructed using Gephi software (version 0.9.2) based on significant Spearman rank correlations (|ρ| ≥ 0.6, *p*<0.05) with multiple testing correction applied. Network topology parameters including node degree, betweenness centrality, and modularity were calculated to characterize community interaction patterns. Only correlations based on genera present in >50% of samples with relative abundance >0.1% were included to ensure statistical reliability.

Microbe-metabolite association analysis employed Spearman correlation analysis between key microbial genera (relative abundance >1%) and major aroma compounds, with statistical significance threshold set at *p*<0.05 after false discovery rate correction. Correlation strength was classified as strong (|ρ| ≥ 0.8), moderate (0.5 ≤ |ρ| < 0.8), or weak (|ρ| < 0.5).

### Statistical Analysis Framework

All statistical analyses incorporated three biological replicates per treatment group to enable robust inference. While three biological replicates per treatment represent a borderline sample size for multivariate analyses, this design is consistent with published tobacco fermentation studies and was supplemented by stringent statistical thresholds, conservative correlation criteria, and multiple testing corrections to minimize false discovery rates. Aroma compound comparisons utilized one-way analysis of variance (ANOVA) followed by Tukey's honestly significant difference (HSD) post-hoc tests for pairwise comparisons. Assumptions of normality and homoscedasticity were verified using Shapiro-Wilk and Levene's tests respectively, with log-transformation applied when necessary.

For multiple testing correction, the Benjamini-Hochberg false discovery rate (FDR) procedure with α = 0.05 was applied to control Type I error inflation when comparing treatment effects across multiple aroma compounds and microbial taxa simultaneously. Spearman correlation coefficients between microbial genera and aroma compounds were calculated with significance levels set at *p* < 0.05 after FDR correction. Correlation strength was classified as strong (|ρ| ≥ 0.8), moderate (0.5 ≤ |ρ| < 0.8), or weak (|ρ| < 0.5), consistent with the classification criteria applied in microbe-metabolite association analyses.

Variable importance in projection (VIP) scores were calculated through partial least squares-discriminant analysis (PLS-DA) to identify compounds with the greatest discriminatory power between treatment groups, with VIP ≥ 1.0 indicating significant contribution to group separation. All statistical analyses were performed using R software (version 4.2.0) with specialized packages for microbiome and metabolomics data analysis.

## Results and Analysis

### Screening and Phylogenetic Characteristics of Aroma-producing Strains

**Fig. 1:**
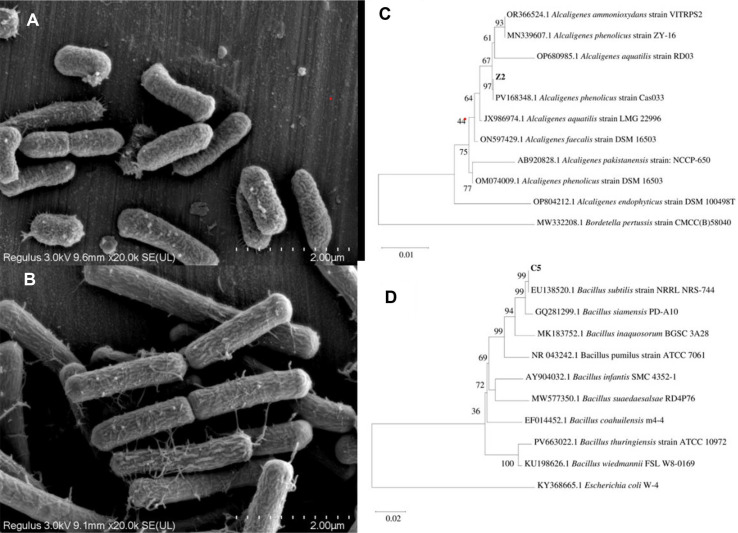
Morphological characteristics and phylogenetic analysis of aroma-producing strains. (**A**) Cell morphology of strain Z2; (**B**) Cell morphology of strain C5; c: Phylogenetic tree of strain Z2; d: Phylogenetic tree of strain C5.

Through standardized olfactory evaluation protocols, two bacterial strains (Z2 and C5) exhibiting distinct aromatic characteristics were isolated from cigar tobacco leaves during the air-curing process. Strain Z2 produced a sweet aroma with rice-like characteristics, while strain C5 generated fruity aromatic notes. Fig. 1 displays the morphological and phylogenetic characteristics of strains Z2 and C5. Morphological examination revealed that both strains exhibited typical rod-shaped bacterial morphology (Fig. 1A and 1B). Phylogenetic analysis based on 16S rRNA gene sequences (Fig. 1C and 1D) indicated 99.2% similarity between strain Z2 and *Alcaligenes phenolicus* reference sequences, while strain C5 showed 98.7% similarity to *Bacillus subtilis*, supporting taxonomic identification as *Alcaligenes phenolicus* Z2 and *Bacillus subtilis* C5. Bootstrap values >90% confirmed the reliability of phylogenetic placements. These taxonomically characterized strains with contrasting aromatic properties provided defined biological materials for subsequent tobacco fermentation studies.

### Comparison of Aroma Compound Composition and Content in Tobacco Leaves

The volatile compound composition of cigar tobacco leaves from different treatments was determined using optimized HS-SPME-GC × GC-MS methodology. From comprehensive analysis of 63 detected compounds across all samples, 20 major aroma compounds were selected based on rigorous statistical criteria, representing diverse chemical classes including alkaloids, diterpenes, fatty alcohols, terpenoids, fatty acid esters, alkenes, alcohols, ketones, furanones, and other organic compounds (Table 1). The experimental design compared single-strain inoculations with Z2 (A. sp.) and C5 (B. sp.), mixed inoculation (M group), and uninoculated controls (CN).

### Statistical Analysis of Compound Variations

GC-MS analysis revealed treatment-specific patterns in aroma compound profiles with statistical significance confirmed by ANOVA (Table 1). Based on variable importance in projection analysis, compounds were ranked by discriminatory power, with nicotine (VIP = 3.014), neophytadiene (VIP = 2.660), and E,E,Z-1,3,12-nonadecatriene-5,14-diol (VIP = 2.432) showing the highest values among the 20 selected major aroma compounds representing diverse chemical classes.

Nicotine concentrations demonstrated the most significant treatment effects, with C5 and M groups showing substantial increases of 1.46-fold and 1.49-fold respectively compared to controls, while Z2 treatment exhibited a modest 1.10-fold increase. This pattern corresponds to nicotine's highest VIP score (3.014) in discriminant analysis, indicating its greatest contribution to treatment group separation.

Neophytadiene showed distinct treatment responses, with Z2 treatment (21.17 ± 0.64) exhibiting a 1.06-fold increase compared to controls (19.91 ± 0.58), while C5 (14.56 ± 1.03) and M groups (14.78 ± 0.54) showed reduced concentrations at 0.73-fold and 0.74-fold respectively. The pronounced treatment differences align with neophytadiene's high VIP value (2.660).

The most pronounced quantitative change occurred in 2-hexyl-1-decanol concentrations, where C5 treatment (1.77 ± 0.34) showed 9.67-fold higher levels than controls (0.18 ± 0.10). Z2 (0.62 ± 0.22) and M treatments (0.88 ± 0.44) showed more moderate but statistically significant increases of 3.40-fold and 4.81-fold respectively. Additional significant changes included 17-pentatriacontene, with M group (3.33 ± 0.15) showing 1.91-fold increases compared to controls (1.74 ± 0.18), while Z2 (1.39 ± 0.29) and C5 (2.07 ± 0.53) treatments showed 0.79-fold and 1.19-fold changes respectively.

### Detection of Treatment-Specific Compounds

Several compounds showed treatment-specific detection patterns. The complex terpenoid 1,1,6-trimethyl-3-methylene-2-cyclohexane derivative was undetected in control samples but appeared in Z2 (3.98 ± 0.27) and M treatments (3.10 ± 0.11), while C5 treatment showed minimal levels (0.72 ± 0.19), suggesting possible strain-specific differences in terpenoid compound accumulation, though the underlying mechanisms remain to be determined. Similarly, linoleyl palmitate was undetected in Z2 samples but present in controls (2.83 ± 0.41), C5 (1.30 ± 0.20), and M groups (1.03 ± 0.26). Additionally, 1,2,3,6-tetrahydro-2,3'-bipyridine was absent in controls but appeared in Z2 (1.19 ± 0.02), C5 (0.52 ± 0.16), and M treatments (0.89 ± 0.13).

### Mixed Inoculation Performance Assessment

The mixed inoculation group showed selective advantages for specific compounds, including 17-pentatriacontene showing the highest response (3.33±0.15, 1.91-fold increase) and nicotine (27.33 ± 1.97, 1.49-fold increase) compared to controls (1.74 ± 0.18 and 18.40 ± 2.22 respectively). However, for the majority of analyzed compounds, mixed inoculation often showed intermediate effects between single-strain treatments, without evidence of additive or enhanced responses, suggesting potential competitive interactions between Z2 and C5 strains that warrant further investigation.

### Multivariate Analysis of Compound Patterns

Statistical analysis revealed distinct treatment-specific aroma profiles based on VIP analysis and compound response patterns: Z2 treatment was primarily associated with elevated diterpene compound concentrations (neophytadiene showing 1.06-fold increase) and specific terpenoids (1,1,6-trimethyl-3-methylene-2-cyclohexane derivative), while C5 treatment was associated with markedly higher alcohol concentrations (2-hexyl-1-decanol showing 9.67-fold increase) and elevated alkaloid levels (nicotine showing 1.46-fold increase). Mixed inoculation showed selective advantages for specific compounds including 17-pentatriacontene (1.91-fold increase) and maintained high nicotine levels (1.49-fold increase). These patterns indicate that exogenous strain addition was associated with measurable alterations to tobacco leaf volatile profiles, with each treatment showing distinct compound accumulation characteristics.

**Fig. 2:**
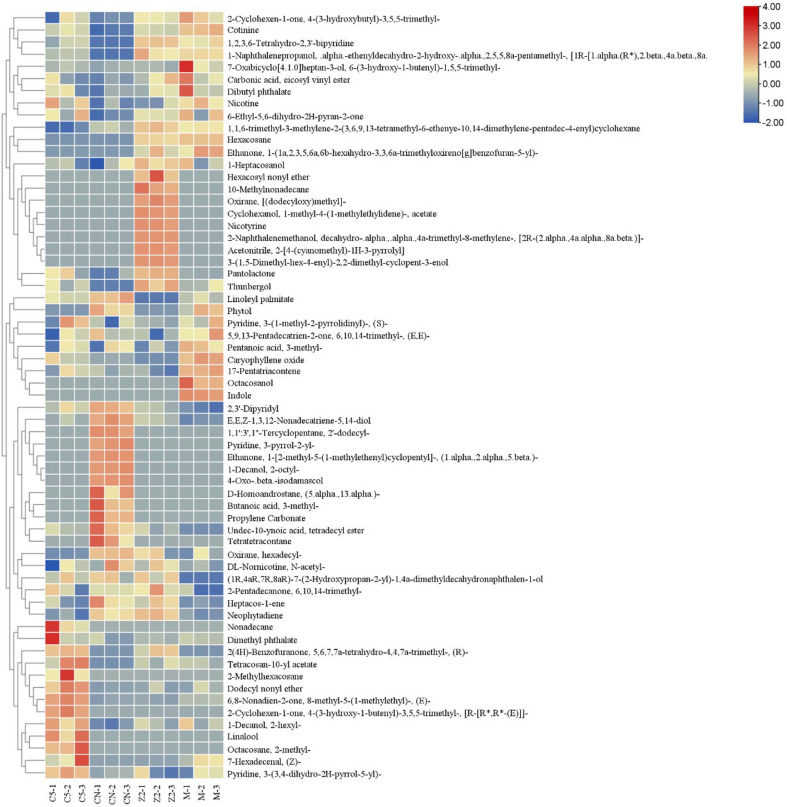
Heat map clustering analysis of aroma compounds in different treatment groups. Color intensity represents log-transformed and z-score normalized relative peak areas. Red indicates relatively higher abundance and blue indicates relatively lower abundance. Rows represent individual compounds and columns represent treatment groups. Hierarchical clustering was performed using Euclidean distance and Ward's linkage algorithm.

Fig. 2 presents hierarchical clustering analysis of all 63 detected compounds across treatment groups. The heat map visualization uses color intensity to represent log-transformed and z-score normalized relative peak areas, with clustering based on Euclidean distance and Ward's algorithm identifying distinct compound clusters with differential treatment responses. The dendrogram structure reveals treatment-specific groupings, with each treatment showing characteristic volatile profiles that align with the discriminatory compounds identified through VIP analysis.

### Microbial Community Structure Analysis

**Fig. 3:**
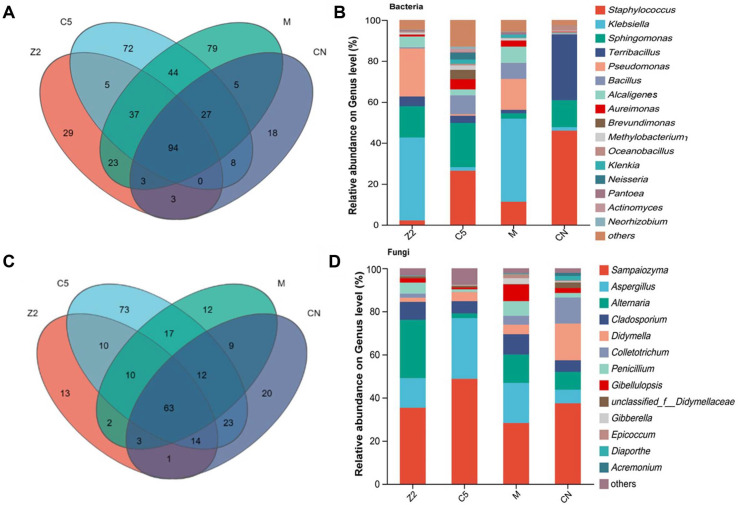
Microbial Venn analysis and community composition at the genus level. (**A**) Bacterial Venn diagram showing shared and unique genera across treatment groups; (**B**) Bacterial community relative abundance composition at the genus level; (**C**) Fungal Venn diagram showing shared and unique genera across treatment groups; (**D**) Fungal community relative abundance composition at the genus level.

High-throughput sequencing analysis of bacterial and fungal communities revealed treatment-associated changes in microbiome composition and structure. Bacterial community analysis identified varying levels of diversity and taxonomic composition across treatments (Fig. 3). Venn diagram analysis showed that treatment groups shared 21% of bacterial genera as core microbiome, with treatment-specific genera representing 6% (Z2), 16% (C5), 18% (M), and 4% (CN) of total diversity. These proportions indicate substantial differences in community membership across treatment groups.

Relative abundance analysis of major bacterial genera (>1% abundance) revealed significant shifts in dominant taxa. Notable increases in Alcaligenes and Bacillus abundance in Z2 and C5 treatments respectively were consistent with the presence of introduced strains, though direct confirmation of strain establishment would require quantitative tracking throughout the fermentation period. Dominant community composition varied substantially: controls were characterized by *Staphylococcus*, *Terribacillus*, and *Sphingomonas*; Z2 treatments by *Klebsiella*, *Pseudomonas*, and *Sphingomonas*; C5 treatments by *Staphylococcus*, *Sphingomonas*, and *Bacillus*; and mixed inoculations by *Klebsiella*, *Staphylococcus*, and *Pseudomonas*.

Fungal community analysis revealed more limited treatment effects. While Sampaiozyma remained the dominant genus across all treatments, Aspergillus showed treatment-associated increases with 1.2, 3.5, and 2.0-fold higher abundance in Z2, C5, and M groups respectively compared to controls.

### Principal Coordinate Analysis of Community Structure

**Fig. 4:**
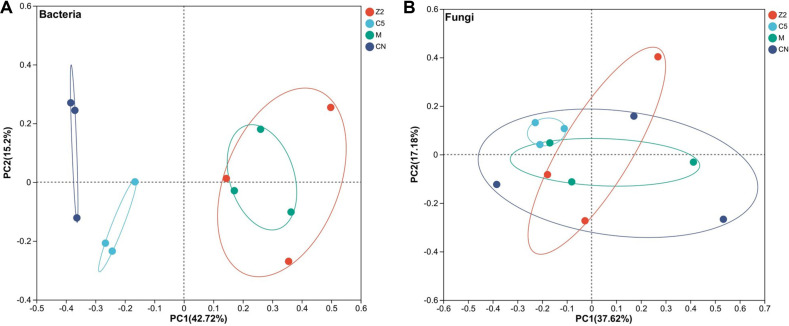
β-diversity principal coordinate analysis. (A) Principal coordinate analysis of bacterial communities; (B) Principal coordinate analysis of fungal communities. Different colors represent different treatment groups: red-Z2 group, cyan-C5 group, green-M group, dark blue-CN group.

PCoA analysis revealed distinct bacterial community structure across treatment groups (Fig. 4). The first two principal coordinates explained 57.92% of total variation (PC1: 42.72%, PC2: 15.20%). PERMANOVA analysis confirmed significant treatment effects (*p* = 0.002). Treatment groups showed distinct clustering patterns along both principal coordinates. C5 treatments clustered with strongly positive PC2 values, Z2 treatments with negative PC2 values, and mixed inoculations occupied intermediate positions, suggesting that bacterial community composition differed distinctly in association with different strain treatments.

Fungal community PCoA showed minimal treatment separation, indicating relatively consistent fungal community composition across treatment groups regardless of bacterial strain inoculation. Consequently, subsequent network and correlation analyses focused on bacterial communities where treatment effects were most pronounced.

### Analysis of Microbial Interaction Network Characteristics

**Fig. 5:**
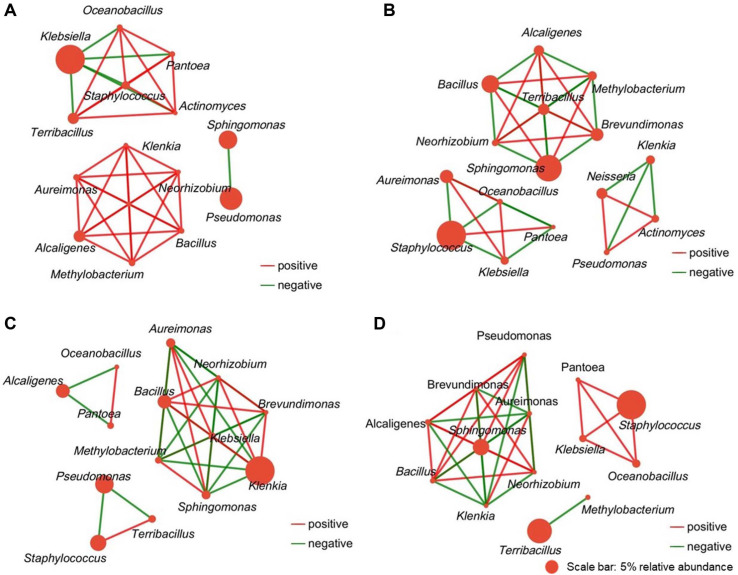
Major bacterial interaction networks based on correlation analysis. (**A**) Z2 group; (**B**) C5 group; (**C**) M group; (**D**) CN group. Red lines represent positive correlations, green lines represent negative correlations. Edge thickness reflects Spearman correlation coefficient strength (|ρ| range: 0.6–1.0), with thicker edges indicating stronger correlations. Node size is proportional to the relative abundance of each bacterial genus, with the scale bar indicating 5% relative abundance. Only statistically significant correlations (|ρ| ≥ 0.6, *p* < 0.05) after multiple testing correction are shown.

To examine potential microbial interaction patterns across treatment groups and their possible associations with aroma compound profiles, co-occurrence networks were constructed based on statistically significant Spearman correlations (|ρ| ≥ 0.6, *p* < 0.05) with multiple comparison correction (Fig. 5). In Fig. 5, node size is proportional to the relative abundance of each genus with scale indicated in the figure legend, and edge thickness reflects the strength of Spearman correlation coefficients. Network analysis revealed distinct correlation patterns among treatment groups, though causal relationships between network properties and metabolic outcomes cannot be established from correlational data alone.

The Z2 treatment group (Fig. 5A) exhibited a network structure with notable positive correlation patterns, forming apparent sub-networks. One cluster included genera such as *Alcaligenes*, *Bacillus*, *Brevundimonas*, *Klenkia*, *Aureimonas*, *Neorhizobium*, and *Methylobacterium*, while another comprised *Staphylococcus*, *Oceanobacillus*, *Pantoea*, *Terribacillus*, and *Actinomyces*. This pattern suggests that Z2 treatment was associated with distinct positive co-occurrence patterns among bacterial taxa, though whether these reflect direct cooperative interactions or shared environmental responses remains unclear.

The C5 treatment network (Fig. 5B) showed a different correlation distribution pattern compared to Z2. Notable positive correlations occurred among *Alcaligenes*, *Brevundimonas*, *Neorhizobium*, and *Terribacillus*, while *Bacillus* showed limited positive associations primarily with *Methylobacterium* and *Sphingomonas*. These observations suggest that C5 treatment was associated with distinct co-occurrence patterns characterized by more selective statistical associations between specific taxa.

Mixed inoculation (M group, Fig. 5C) demonstrated a network with distinct correlation characteristics, involving genera including *Bacillus*, *Klebsiella*, *Neorhizobium*, *Brevundimonas*, *Sphingomonas*, *Klenkia*, *Methylobacterium*, and *Aureimonas*. Positive correlations were primarily observed among *Bacillus*, *Klebsiella*, *Neorhizobium*, and *Brevundimonas*. The network structure was consistent with patterns that might reflect competitive interactions, though alternative explanations including shared substrate preferences or environmental responses cannot be excluded.

Control treatment networks (CN group, Fig. 5D) showed stable positive associations between *Staphylococcus*, *Oceanobacillus*, *Pantoea*, and *Klebsiella*, as well as among *Bacillus*, *Alcaligenes*, *Brevundimonas*, *Neorhizobium*, *Sphingomonas*, and *Pseudomonas*, representing the baseline microbial co-occurrence patterns in uninoculated tobacco samples.

### Interpretive Limitations of Network Analysis

While the observed network patterns suggest distinct microbial co-occurrence characteristics across treatment groups, direct causal links between network topology and metabolic outcomes cannot be established from correlational data alone. The observed patterns may reflect: (1) direct microbial interactions, (2) shared responses to environmental conditions, such as temperature-modulated biochemical pathways that influence both microbial communities and metabolite profiles [15], (3) indirect effects mediated through substrate competition or metabolite exchange, or (4) statistical artifacts from compositional data constraints. Future studies employing controlled co-culture experiments and metabolic modeling would be needed to validate proposed interaction mechanisms and establish the relationship between community structure and aroma compound production, particularly given the complex physicochemical properties and diverse aroma compound compositions observed across different cigar tobacco varieties [16].

### Correlation Analysis between Microorganisms and Aroma Compounds

Spearman correlation analysis was performed between major bacterial genera (relative abundance >1%) and key aroma compounds to identify potential associations across treatment groups (Table 2). The analysis focused on compounds showing high variable importance in projection scores and significant treatment effects, including nicotine, neophytadiene, 2-hexyl-1-decanol, and 3-(1-methyl-2-pyrrolidinyl)-pyridine. This analysis aimed to explore possible relationships between microbial community composition and volatile compound profiles, while recognizing that correlation does not establish causation.

### Treatment-Specific Association Patterns

In Z2-treated samples, *Klebsiella* (r = 0.892, *p* < 0.05) and *Pseudomonas* (r = 0.856, *p* < 0.05) both showed strong positive associations with 3-(1-methyl-2-pyrrolidinyl)-pyridine, suggesting potential associations with alkaloid compound accumulation, though the mechanistic basis for these relationships requires further investigation. These associations may reflect direct or indirect effects of community composition changes, though the specific mechanisms cannot be determined from correlational data alone. Alcaligenes showed a positive trend with neophytadiene (r = 0.734), though this association did not reach statistical significance after correction and should be interpreted with caution.

C5 treatment samples showed notable patterns with Bacillus demonstrating strong negative associations with neophytadiene (r = -0.823, *p* < 0.05) and strong positive associations with 2-hexyl-1-decanol (r = 0.891, *p* < 0.01). These contrasting patterns suggest a possible negative association between Bacillus abundance and neophytadiene levels alongside a positive association with 2-hexyl-1-decanol, though whether this reflects direct metabolic activity or indirect community-level effects remains speculative and requires experimental validation. *Staphylococcus* showed a positive trend with nicotine (r = 0.745), though this association did not reach statistical significance after correction.

Mixed inoculation samples generally exhibited reduced association strength compared to single-strain treatments. Alcaligenes showed substantially diminished correlations with neophytadiene (r = 0.432, NS), while *Klebsiella* showed moderate correlations with nicotine (r = 0.623, NS), both non-significant after correction. These patterns are consistent with the hypothesis of competitive interactions in mixed inoculation, though alternative explanations cannot be excluded.

Control samples showed minimal correlations between indigenous genera (Terribacillus r = 0.245, NS; Sphingomonas r = 0.318, NS) and baseline aroma compounds. This baseline pattern contrasts with the stronger associations observed in inoculated treatment groups, suggesting that exogenous strain addition may be associated with altered microbe-metabolite co-occurrence patterns, though causal relationships require further experimental validation.

### Statistical Assessment and Pattern Recognition

The observed correlation patterns demonstrated treatment-specific differences in microbe-metabolite associations, with single-strain treatments (Z2 and C5) generally showing stronger and more statistically significant correlations compared to mixed inoculation and control groups. Z2 treatment exhibited strong associations between specific genera (*Klebsiella*, *Pseudomonas*) and alkaloid compounds, while C5 treatment showed distinct patterns with Bacillus correlations. Mixed inoculation groups demonstrated reduced correlation strength, with most associations becoming non-significant, consistent with potential competitive effects. These patterns align with the aroma compound abundance data and VIP analysis results, providing preliminary evidence for strain-specific microbe-metabolite associations in tobacco fermentation, though causal contributions require further experimental validation [17].

### Future Validation Requirements

The correlation analyses establish a foundation for understanding potential microbial contributions to tobacco aroma development and provide preliminary insights for biotechnology applications in fermentation optimization. However, the correlational nature of these findings requires validation through controlled mechanistic studies. Future research should focus on: (1) controlled single-strain metabolic assays to establish direct metabolic capabilities, (2) temporal sampling to capture dynamic microbe-metabolite interactions, (3) functional genomic analysis to identify specific metabolic pathways, and (4) validation across different tobacco varieties and environmental conditions. These validation steps are essential before developing standardized microbial inoculation protocols for enhanced tobacco fermentation.

## Discussion

### Potential Mechanisms of Aroma Enhancement by Exogenous Strains

Through optimization of HS-SPME-GC×GC-MS detection conditions, this study identified 63 volatile compounds in total, from which 20 major aroma compounds were selected based on rigorous statistical criteria including statistical significance (*p* < 0.05), variable importance in projection scores with priority for VIP ≥ 1.0, and chemical class representation to ensure comprehensive aroma profile coverage.

The introduction of exogenous strains Z2 (*A. phenolicus*) and C5 (*B. subtilis*) was associated with measurable alterations to aroma compound profiles across multiple chemical classes (Table 1). Notably, nicotine showed the most pronounced treatment-associated differences, with C5 and M groups showing substantially higher levels compared to controls, while neophytadiene exhibited contrasting patterns between Z2 and C5 treatments. The most striking quantitative difference involved 2-hexyl-1-decanol, which showed markedly elevated concentrations in C5-treated samples, alongside notable increases in 17-pentatriacontene in mixed inoculation samples. These treatment-associated differences in volatile compound accumulation suggest strain-specific influences on the tobacco leaf chemical environment, though the underlying mechanisms warrant further investigation through controlled metabolic studies.

### Hypothetical Metabolic Mechanisms

The observed changes may involve multiple potential mechanisms, though direct causal relationships remain to be established. Alcaligenes species have been reported to possess diverse metabolic capabilities including fatty acid metabolism and aromatic compound biotransformation [18]. The higher levels of neophytadiene, certain long-chain alcohols, and nitrogen-containing compounds observed in Z2-treated samples may be consistent with such metabolic capabilities, though direct enzymatic evidence would be required for confirmation.

Similarly, Bacillus strains are known to harbor complex secondary metabolic pathways and polysaccharide-degrading enzyme systems. [19]. The higher concentrations of 2-hexyl-1-decanol and other alcohols observed in C5-treated samples have been hypothesized to potentially involve lipid metabolism or plant cell wall degradation pathways, though this interpretation remains speculative without direct biochemical validation.

### Limitations of Mechanistic Inferences

The associations observed between strain additions and compound changes should be interpreted cautiously. These associations may reflect: (1) direct metabolic contributions by introduced strains, (2) indirect effects through modulation of indigenous microbiota, (3) altered plant metabolic responses to microbial presence, or (4) combinations of these factors [20]. For instance, while Z2 treatment was associated with detection of specific terpenoid derivatives (such as the 1,1,6-trimethyl-3-methylene-2-cyclohexane derivative) that were previously absent, whether this reflects novel biosynthetic activity or enhanced extraction/stability requires targeted metabolomic investigation.

The possibility that strain Z2 may be associated with carotenoid degradation pathway activities, while plausible based on observed compound patterns, remains speculative without direct enzyme activity measurements or pathway-specific gene expression analysis. Future studies should employ isotope labeling experiments, purified enzyme assays, and transcriptomic approaches to validate proposed mechanistic links between microbial activities and observed chemical changes [21].

### Microbial Interaction Patterns and Their Potential Role in Tobacco Fermentation

Network analysis revealed distinct microbial co-occurrence patterns across treatment groups that may be associated with differential fermentation outcomes. The Z2 treatment group exhibited notable positive co-occurrence patterns among bacterial taxa, while mixed inoculation showed different network characteristics consistent with patterns that might reflect competitive interactions, though alternative explanations including shared environmental responses cannot be excluded.

### Interpretive Cautions for Network Data

While these correlation patterns are intriguing, several important limitations must be acknowledged. Co-occurrence networks derived from amplicon sequencing data reflect statistical associations rather than direct ecological interactions. The observed patterns could result from: (1) genuine cooperative or competitive relationships, (2) shared environmental preferences, (3) indirect effects mediated through substrate utilization, or (4) technical artifacts from sequencing depth variations.

The suggestion that positive microbial co-occurrence patterns may be associated with enhanced aroma compound accumulation requires validation through controlled experiments. Establishing causality would require: (1) controlled co-culture studies with defined media, (2) metabolite exchange experiments to identify specific interaction compounds, (3) temporal sampling to capture interaction dynamics, and (4) functional genomic analysis of interaction mechanisms [22-23].

### Alternative Explanations for Observed Patterns

The association between network topology and aroma enhancement could alternatively reflect: (1) Z2's superior colonization ability leading to stable community establishment, (2) production of signaling molecules that reduce competitive stress, (3) metabolic complementarity reducing resource competition, or (4) indirect effects through pH or nutrient modifications [24]. Distinguishing among these possibilities requires targeted experimental approaches beyond the scope of correlational analysis.

### Comparative Assessment of Single versus Mixed Inoculation Strategies

This study provides preliminary observations suggesting potential advantages of single-strain over mixed inoculation approaches for cigar tobacco leaf aroma enhancement. Single-strain treatments generally showed more pronounced and treatment-specific compound associations compared to mixed applications, though the underlying causes remain speculative.

Several non-exclusive explanations may account for the intermediate or reduced effects observed in mixed inoculation: (1) direct competitive interactions between Z2 and C5 strains, (2) incompatible environmental requirements or metabolic byproducts, (3) disrupted establishment patterns due to strain interference, or (4) altered interactions with indigenous microbiota. Network analysis revealed distinct co-occurrence patterns in mixed inoculation samples consistent with potential competitive dynamics, though alternative explanations including differential establishment kinetics or disrupted indigenous community responses cannot be excluded [25].

From practical perspectives, single strain approaches offer potential advantages including simplified quality control, predictable outcomes, and targeted aroma development. Based on observed associations, strain selection might be tailored to desired aroma profiles: *A*. sp. Z2 was associated with higher terpene compound concentrations, while *B*. sp. C5 was associated with elevated alcohol and alkaloid levels. However, this approach requires validation across different tobacco varieties, environmental conditions, and fermentation scales before any commercial implementation can be considered [26].

### Limitations and Future Research Directions

This study's findings are constrained by several methodological limitations: (1) amplicon sequencing provides taxonomic but limited functional information, (2) single time-point sampling cannot capture temporal dynamics, (3) correlation-based inferences cannot establish causation, and (4) laboratory conditions may not reflect industrial fermentation environments. While this study demonstrates measurable associations between bacterial strain additions and tobacco leaf volatile profiles and microbial community structure, the mechanisms underlying these changes remain largely speculative. The observed patterns provide valuable hypotheses for future mechanistic research but should not be interpreted as definitive evidence for specific metabolic or ecological processes.

Future research should prioritize: (1) functional metagenomics to characterize metabolic capabilities, (2) time-series sampling to understand succession dynamics, (3) controlled metabolic studies with purified strains, (4) enzyme activity assays for proposed pathways, (5) scale-up trials under commercial conditions, and (6) economic analysis of implementation feasibility. Translation to commercial applications requires substantial additional validation across different conditions, scales, and tobacco varieties.

### Potential for Microbiomics-Based Fermentation Optimization

This study suggests possible microbiomics applications for tobacco fermentation improvement, though significant development is needed before practical implementation. Traditional approaches controlling physical parameters show variable effectiveness, potentially creating opportunities for microbiome-guided strategies. Potential future improvements could include: strain library development with characterized aroma profiles, microbiome-informed selection of compatible strain combinations, and dynamic monitoring systems tracking microbial and metabolic indicators.

Process enhancement should consider colonization stability through pre-treatment protocols, optimized inoculation parameters, and strain-specific substrate formulations. Advanced biotechnologies including metabolic engineering could potentially enhance strain capabilities, though regulatory and safety considerations would require careful evaluation. However, these microbiomics approaches face substantial challenges including strain stability, environmental variability, regulatory approval, and economic feasibility [27].

The proposed systematic optimization represents a long-term research goal rather than immediate commercial application. While microbiomics offers promising directions for fermentation control and customization, translation from laboratory findings to industrial practice demands comprehensive development and validation efforts, as detailed in the limitations section above.

## Conclusion

Two aroma-producing strains, *Alcaligenes phenolicus* Z2 and *Bacillus subtilis* C5, isolated from cigar tobacco leaf curing processes, demonstrated measurable associations with volatile compound profiles in tobacco leaves through single strain or mixed inoculation approaches. Analysis of volatile compounds indicated that microbial treatments were associated with treatment-specific differences in aroma compound compositions, with each approach showing distinct patterns across different compound classes. Microbial co-occurrence network analysis suggested that different strain treatments were associated with varying indigenous community interaction patterns. Z2 treatment was associated with distinct microbial co-occurrence patterns, which co-occurred with observed differences in compound profiles, though whether any relationship exists between these patterns requires further investigation.

This research contributes to the functional strain collection derived from tobacco sources and provides preliminary evidence for strain-based fermentation strategies. The findings suggest potential applications for reducing reliance on external aroma additives during fermentation, though substantial additional research is needed before commercial implementation. While the results offer useful insights for tobacco fermentation optimization, the mechanisms underlying observed effects remain largely speculative and require validation through controlled mechanistic studies, scale-up trials, and economic feasibility assessment before practical application.

## Supplemental Materials

Supplementary data for this paper are available on-line only at http://jmb.or.kr.



## Figures and Tables

**Table 1:** Concentrations of 20 major aroma compounds in cigar tobacco leaves under different treatments.

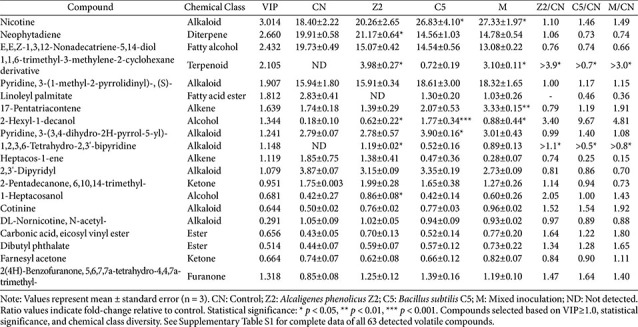

**Table 2:** Correlation patterns between major microorganisms and aroma compounds within treatment groups.