# Supplementary material for: Differential Enhancement of Cigar Tobacco Leaf Aroma by Single-Strain Inoculation with Alcaligenes phenolicus and Bacillus subtilis
Source: J Microbiol Biotechnol. 2026 May 12;36:e2601012. doi: 10.4014/jmb.2601.01012 (PMC13181313; doi:10.4014/jmb.2601.01012)
Supplement: Supplementary file 1 [file jmb-36-e2601012-supple.pdf]

J. Microbiol. Biotechnol. 2026. 36: e2601012

https://doi.org/10.4014/jmb.2601.01012

Table S1. Complete volatile compound profiles detected in cigar tobacco leaves across all treatment groups by HS-SPME-GC×GC-MS analysis.

No	Compound	Chemical Class	VIP	CN	Z2	C5	M	Z2/CN	C5/CN	M/CN
1	Nicotine	Alkaloid	3.014	18.40±2.22	20.26±2.65	26.83±4.10	27.33±1.97	1.10	1.46	1.49
2	Neophytadiene	Alkene	2.660	19.91±0.58	21.17±0.64	14.56±1.03	14.78±0.54	1.06	0.73	0.74
3	E,E,Z-1,3,12-Nonadecatriene-5,14-diol	Alcohol	2.432	19.73±0.49	15.07±0.42	14.54±0.56	13.08±0.22	0.76	0.74	0.66
4	1,1,6-trimethyl-3-methylene-2-cyclohexane derivative	Alkane	2.105	ND	3.98±0.27	0.72±0.19	3.10±0.11	-	-	-
5	Pyridine, 3-(1-methyl-2-pyrrolidinyl)-, (S)-	Alkaloid	1.907	15.94±1.80	15.91±0.34	18.61±3.00	18.32±1.65	1.00	1.17	1.15
6	Linoleyl palmitate	Ester	1.812	2.83±0.41	ND	1.30±0.20	1.03±0.26	-	0.46	0.36
7	17-Pentatriacontene	Alkene	1.639	1.74±0.18	1.39±0.29	2.07±0.53	3.33±0.15	0.79	1.19	1.91
8	2-Hexyl-1-decanol	Alcohol	1.344	0.18±0.10	0.62±0.22	1.77±0.34	0.88±0.44	3.40	9.67	4.81
9	2(4H)-Benzofuranone, 5,6,7,7a-tetrahydro-4,4,7a-trimethyl-	Furanone	1.318	0.85±0.08	1.25±0.12	1.39±0.16	1.19±0.10	1.47	1.64	1.40
10	Pyridine, 3-(3,4-dihydro-2H-pyrrol-5-yl)-	Alkaloid	1.241	2.79±0.07	2.78±0.57	3.90±0.16	3.01±0.43	0.99	1.40	1.08
11	1,2,3,6-Tetrahydro-2,3'-bipyridine	Alkaloid	1.148	ND	1.19±0.02	0.52±0.16	0.89±0.13	-	-	-
12	Heptacos-1-ene	Alkene	1.119	1.85±0.75	1.38±0.41	0.47±0.36	0.28±0.07	0.74	0.25	0.15
13	2,3'-Dipyridyl	Alkaloid	1.079	3.87±0.07	3.15±0.09	3.35±0.19	2.73±0.09	0.81	0.86	0.70
14	Tetracosan-10-yl acetate	Ester	0.991	ND	0.24±0.10	0.74±0.33	0.21±0.06	-	-	-
15	Octacosanol	Alcohol	0.982	ND	ND	ND	0.52±0.16	ND/ND	ND/ND	-
16	2-Pentadecanone, 6,10,14-trimethyl-	Ketone	0.951	1.75±0.003	1.99±0.28	1.65±0.38	1.27±0.26	1.14	0.94	0.73
17	Thunbergol	Alcohol	0.945	ND	0.73±0.09	0.33±0.12	0.31±0.12	-	-	-
18	1-Decanol, 2-octyl-	Alcohol	0.943	0.75±0.03	ND	ND	ND	ND	ND	ND
19	Nicotyrine	Alkaloid	0.930	ND	0.58±0.02	ND	ND	-	ND/ND	ND/ND
20	Oxirane, hexadecyl-	Alkane	0.919	1.00±0.04	0.58±0.35	0.06±0.01	0.30±0.27	0.58	0.06	0.30
21	Ethanone, 1-(1a,2,3,5,6a,6b-hexahydro-3,3,6a-trimethyloxireno[g]benzofuran-5-yl)-	Ketone	0.763	ND	0.24±0.10	ND	0.36±0.10	-	ND/ND	-

22	Octacosane, 2-methyl-	Alkane	0.745	ND	ND	0.32±0.10	ND	ND/ND	-	ND/ND
23	Acetonitrile, 2-[4-(cyanomethyl)-1H-3-pyrrolyl]	Nitrile	0.720	ND	0.35±0.01	ND	ND	-	ND/ND	ND/ND
24	1,1':3',1''-Tercyclopentane, 2'-dodecyl-	Alkane	0.720	0.44±0.03	ND	ND	ND	ND	ND	ND
25	Indole	Indole	0.696	ND	ND	ND	0.253±0.012	ND/ND	ND/ND	-
26	D-Homoandrostane, (5.alpha.,13.alpha.)-	Alkane	0.691	0.43±0.17	ND	ND	ND	ND	ND	ND
27	Hexacosane	Alkane	0.690	ND	0.23±0.01	ND	0.27±0.01	-	ND/ND	-
28	1-Heptacosanol	Alcohol	0.681	0.42±0.27	0.86±0.08	0.42±0.14	0.60±0.26	2.05	1.00	1.43
29	Caryophyllene oxide	Alkene	0.675	0.06±0.01	ND	0.19±0.07	0.37±0.04	ND	2.98	6.03
30	6-Ethyl-5,6-dihydro-2H-pyran-2-one	Ketone	0.674	ND	0.61±0.02	0.65±0.22	0.70±0.27	-	-	-
31	Farnesyl acetone	Ketone	0.664	0.74±0.07	0.62±0.08	0.66±0.12	0.82±0.07	0.84	0.90	1.11
32	Carbonic acid, eicosyl vinyl ester	Ester	0.656	0.43±0.05	0.70±0.13	0.52±0.14	0.77±0.20	1.64	1.22	1.80
33	Dimethyl phthalate	Ester	0.650	0.37±0.08	0.39±0.01	0.679±0.33	0.43±0.02	1.07	1.86	1.19
34	Tetratetracontane	Alkane	0.646	0.39±0.18	ND	ND	ND	ND	ND	ND
35	Cotinine	Alkaloid	0.644	0.50±0.02	0.76±0.02	0.77±0.03	0.96±0.02	1.52	1.54	1.92
36	(1R,4aR,7R,8aR)-7-(2-Hydroxypropan-2-yl)-1,4a-dimethyldecahydronaphthalen-1-ol	Alcohol	0.613	0.25±0.10	0.27±0.07	0.22±0.08	ND	1.07	0.87	ND
37	1-Naphthalenepropanol, .alpha.-ethenyldecahydro-2-hydroxy-.alpha.,2,5,5,8a-pentamethyl-, [1R-[1.alpha.(R*),2.beta.,4a.beta.,8a.]]	Alcohol	0.601	ND	0.31±0.06	0.16±0.01	0.30±0.01	-	-	-
38	Dodecyl nonyl ether	Ether	0.579	0.11±0.01	0.14±0.04	0.34±0.05	0.15±0.04	1.24	3.04	1.37
39	3-(1,5-Dimethyl-hex-4-enyl)-2,2-dimethyl-cyclopent-3-enol	Alcohol	0.577	ND	0.23±0.01	ND	ND	-	ND/ND	ND/ND
40	6,8-Nonadien-2-one, 8-methyl-5-(1-methylethyl)-, (E)-	Ketone	0.554	0.19±0.01	0.19±0.01	0.39±0.02	0.23±0.01	1.01	2.06	1.20
41	Phytol	Alcohol	0.532	0.13±0.02	ND	ND	0.11±0.04	ND	ND	0.86
42	Dibutyl phthalate	Ester	0.514	0.44±0.07	0.59±0.07	0.57±0.12	0.73±0.22	1.34	1.28	1.65
43	2-Cyclohexen-1-one, 4-(3-hydroxy-1-butenyl)-3,5,5-trimethyl-, [R-[R*,R*-(E)]]-	Ketone	0.508	ND	ND	0.14±0.02	ND	ND/ND	-	ND/ND
44	4-Oxo-.beta.-isodamascol	Alcohol	0.490	0.20±0.01	ND	ND	ND	ND	ND	ND
45	Ethanone, 1-[2-methyl-5-(1-methylethenyl)cyclopentyl]-, (1.alpha.,2.alpha.,5.beta.)-	Ketone	0.443	0.17±0.00	ND	ND	ND	ND	ND	ND
46	Nonadecane	Alkane	0.425	ND	ND	0.11±0.07	ND	ND/ND	-	ND/ND
47	Undec-10-ynoic acid, tetradecyl ester	Ester	0.406	0.19±0.05	0.05±0.03	0.07±0.02	ND	0.29	0.36	ND
48	7-Hexadecenal, (Z)-	Aldehyde	0.375	ND	ND	0.14±0.09	0.08±0.04	ND/ND	-	-

49	Cyclohexanol, 1-methyl-4-(1-methylethylidene)-, acetate	Alcohol	0.369	ND	0.09±0.00	ND	ND	-	ND/ND	ND/ND
50	Hexacosyl nonyl ether	Ether	0.369	ND	0.10±0.03	ND	ND	-	ND/ND	ND/ND
51	2-Naphthalenemethanol, decahydro-.alpha.,.alpha.,4a-trimethyl-8-methylene-, [2R-(2.alpha.,4a.alpha.,8a.beta.)]-	Alcohol	0.355	ND	0.09±0.00	ND	ND	-	ND/ND	ND/ND
52	2-Methylhexacosane	Alkane	0.340	ND	ND	0.07±0.05	ND	ND/ND	-	ND/ND
53	7-Oxabicyclo[4.1.0]heptan-3-ol, 6-(3-hydroxy-1-butenyl)-1,5,5-trimethyl-	Alcohol	0.335	0.13±0.01	0.14±0.00	0.14±0.01	0.22±0.07	1.05	1.09	1.63
54	10-Methylnonadecane	Alkane	0.328	ND	0.07±0.01	ND	ND	-	ND/ND	ND/ND
55	Pyridine, 3-pyrrol-2-yl-	Pyrrole	0.323	0.09±0.01	ND	ND	ND	ND	ND	ND
56	DL-Nornicotine, N-acetyl-	Alkaloid	0.291	1.05±0.09	1.02±0.05	0.94±0.09	0.93±0.02	0.97	0.89	0.88
57	Pantolactone	Ketone	0.277	0.03±0.01	0.08±0.00	0.06±0.02	0.04±0.00	2.76	1.97	1.34
58	2-Cyclohexen-1-one,4-(3-hydroxybutyl)-3,5,5-trimethyl-	Ketone	0.275	0.15±0.00	0.20±0.00	0.18±0.04	0.22±0.02	1.37	1.20	1.50
59	Propylene Carbonate	Ester	0.232	0.05±0.01	ND	ND	ND	ND	ND	ND
60	Linalool	Alcohol	0.195	ND	ND	0.02±0.01	ND	ND/ND	-	ND/ND
61	Pentanoic acid, 3-methyl-	Acid	0.187	0.07±0.02	0.06±0.01	0.07±0.02	0.09±0.01	0.85	0.97	1.28
62	Oxirane, [(dodecyloxy)methyl]-	Alkane	0.174	ND	0.02±0.00	ND	ND	-	ND/ND	ND/ND
63	Butanoic acid, 3-methyl-	Acid	0.151	0.02±0.01	ND	ND	ND	ND	ND	ND

Note: Values represent mean ± standard error (n=3). CN: Control; Z2: *Alcaligenes phenolicus* Z2; C5: *Bacillus subtilis* C5; M: Mixed inoculation; ND: Not detected; ND/ND: both treatment and control not detected. Ratio values indicate fold-change relative to control. VIP: Variable importance in projection score calculated by PLS-DA. Compounds are listed in descending order of VIP score. Compounds included in Table 1 were selected based on three pre-specified criteria: VIP≥1.0, statistical significance ($p<0.05$ by one-way ANOVA with Tukey's HSD), and chemical class representation. Statistical significance annotations for selected compounds are provided in Table 1.
